# Beyond NMDA Receptors: A Narrative Review of Ketamine’s Rapid and Multifaceted Mechanisms in Depression Treatment

**DOI:** 10.3390/ijms252413658

**Published:** 2024-12-20

**Authors:** Zuzanna Antos, Xawery Żukow, Laura Bursztynowicz, Piotr Jakubów

**Affiliations:** Department of Paediatric Anaesthesiology and Intensive Therapy with Pain Division, Faculty of Medicine, Medical University of Bialystok, 15-089 Bialystok, Poland; 39615@student.umb.edu.pl (Z.A.); 39714@student.umb.edu.pl (X.Ż.); 39843@student.umb.edu.pl (L.B.)

**Keywords:** ketamine, depression, antidepressant, NMDA antagonist, mechanism of action, neuroplasticity, BDNF, triple network dysfunction, opioid system

## Abstract

The rising prevalence of depression, with its associated suicide risk, demands effective fast-acting treatments. Ketamine has emerged as promising, demonstrating rapid antidepressant effects. While early studies show swift mood improvements, its precise mechanisms remain unclear. This article aims to compile and synthesize the literature on ketamine’s molecular actions. Ketamine primarily works by antagonizing NMDA receptors, reducing GABAergic inhibition, and increasing glutamate release. This enhanced glutamate activates AMPA receptors, triggering crucial downstream cascades, including BDNF-TrkB and mTOR pathways, promoting synaptic proliferation and regeneration. Moreover, neuroimaging studies have demonstrated alterations in brain networks involved in emotional regulation, including the Default Mode Network (DMN), Central Executive Network (CEN), and Salience Network (SN), which are frequently disrupted in depression. Despite the promising findings, the literature reveals significant inaccuracies and gaps in understanding the full scope of ketamine’s therapeutic potential. For instance, ketamine engages with opioid receptors, insinuating a permissive role of the opioid system in amplifying ketamine’s antidepressant effects, albeit ketamine does not operate as a direct opioid agonist. Further exploration is requisite to comprehensively ascertain its safety profile, long-term efficacy, and the impact of genetic determinants, such as BDNF polymorphisms, on treatment responsiveness.

## 1. Introduction

Major depressive disorder (MDD) is one of the most common and severe mental health problems in the world, affecting more than 280 million people, or about 3.8% of the population, including 5% of adults [1]. It is characterized by a recurrent and chronic course and is associated with a high risk of suicidal thoughts and attempts [2,3,4].

More than 800,000 deaths by suicide are attributed to it each year [5,6].

This disorder is one of the leading causes of disability with an estimated lifetime prevalence of 15%. Its effects are not only individual but also social and economic, causing the impact on health burden to increase at an alarming rate [7,8]. According to the World Health Organization (WHO), it will rank first in the global disease burden by 2030 [9]. In addition, two-thirds of patients with MDD do not respond to first-line treatment [10]. Approximately 30% of patients fail to respond to at least two classic antidepressants at the appropriate dose and duration (≥6 weeks), a condition referred to as treatment-resistant depression (TRD) [11]. Moreover, despite the resolution of depressive symptoms in some patients, 25–60% of them experience relapse [12].

Contemporary antidepressant treatment mainly focuses on the monoamine serotonin (5-HT), norepinephrine (NE), and dopamine (DA) deficiency hypothesis, accepting it as a critical driver of depression [13,14,15]. Conventional antidepressants, such as tricyclic antidepressants (TCAs), monoamine oxidase inhibitors (MAOIs), selective serotonin reuptake inhibitors (SSRIs), and norepinephrine reuptake inhibitors (SNRIs), act by regulating monoamines in the central nervous system, increasing serotonergic and noradrenergic neurotransmission [16].

However, their significant limitation is their delayed onset of action and effectiveness. They require several weeks to months (usually 4–6 weeks) of daily intake to induce substantial clinical improvement. During this time, they can exacerbate pre-existing symptoms and suicidal tendencies, especially in young people [17,18].

Therefore, treatment often does not result in significant improvements in quality of life, and for most people, it does not return to normal, even though the mood disorder is alleviated. The ineffectiveness of this drug therapy may demoralize people with MDD, contributing to their increased risk of suicidal thoughts and behavior. This demoralization is likely related to dysfunction of the hypothalamic-pituitary-adrenal axis during stress, resulting in neurogenic inflammation, glutamatergic excitotoxicity, and disruption of neuronal processes [19].

Due to the above limitations in the effectiveness of monoamine antidepressants, the search for new therapeutic solutions has begun. Other neurotransmitters, such as acetylcholine, glutamate, and gamma-aminobutyric acid (GABA), have also been recognized to be involved in the etiology of depression [20].

SSRIs, including fluoxetine and sertraline, have an affinity for 5-HT2 and 5-HT1A receptors [21]. They inhibit presynaptic serotonin reuptake at the serotonin transporter (SERT), resulting in increased neurotransmitter levels at the postsynaptic membrane of serotonergic synapses [22].

In contrast, the goal of MAOIs, for example, selegiline and moclobemide, is to inhibit the enzyme monoamine oxidase, located at the presynaptic end and responsible for the breakdown of biogenic amines [23,24].

On the other hand, TCAs, which include amitriptyline and desipramine, act by blocking presynaptic norepinephrine reuptake transporters and presynaptic serotonin reuptake transporters. In addition, they are antagonists of postsynaptic α1 and α2-adrenergic receptors, postsynaptic muscarinic receptors, and postsynaptic histamine H1 receptors [24,25,26,27].

SNRIs, duloxetine, and venlafaxine have a mechanism of action similar to TCAs. They inhibit 5-HT and NE reuptake at their respective transporters but show little or no effect on adrenergic (α1, α2, and β), histamine (H1), muscarinic, dopaminergic, or postsynaptic 5-HT receptors [28,29].

Other drug groups that have effects on neurotransmitter transporters include selective norepinephrine reuptake inhibitors (NRIs), such as reboxetine and nisoxetine, which block the norepinephrine transporter (NET), and NE reuptake inhibitors (NDRIs), bupropion, that show binding affinity for DA and NE transporters [20,29].

In contrast, serotonin-2 antagonists and reuptake inhibitors (SARIs), trazodone and nefazodone, interact with the α1-adrenergic receptor, resulting in the inhibition of 5-HT and NE reuptake [30], while specific serotonergic antidepressants (NASSAs), including mirtazapine, are antagonists of presynaptic α2-adrenergic autoreceptors and selectively inhibit 5-HT2 and 5-HT3 [27,31].

Also, mianserin, which belongs to the tetracyclic antidepressants (TeCAs), and trazodone, which belongs to SARI, block presynaptic α2 autoreceptors [32].

A new class of antidepressants called multimodal antidepressants (MMAs), of which vilazodone and vortioxetine are representatives, show high affinity for binding multiple 5-HT receptors (such as 5-HT1A, 5-HT1B, 5-HT3A, 5-HT7) [33,34].

In addition, some drugs, such as buspirone and tandospirone, have been approved for the treatment of depression due to their agonism to the 5-HT1A receptor [20,35].

Ketamine has begun to be a target for research due to its different molecular mechanisms and effects on other receptors compared to classic antidepressants. To better highlight ketamine’s unique properties, we first provide a brief overview of commonly used antidepressant drugs in the following paragraph (see Table 1).

Many studies have shown that N-methyl-D-aspartate acid glutamate receptors (NMDARs) play a role in the pathophysiology of MDD and the mechanism of action of antidepressant therapy [38,39,40].

This discovery led to the investigation of ketamine, a drug commonly used as an anesthetic and analgesic [38]. While the precise mechanism of ketamine is not fully understood, it is primarily believed to function as an NMDA receptor antagonist, influencing various neural pathways and neurotransmitter systems. This complexity underscores its therapeutic potential and highlights the challenges associated with its use [41].

A unique aspect of ketamine’s action is its rapid onset, which makes it particularly valuable in emergencies requiring urgent symptom relief, such as severe suicidal ideation or other crises demanding a fast improvement in the patient’s mental state [42,43].

Ketamine can alleviate suicidal thoughts within just 1–2 h of initiating the infusion, offering a crucial lifeline for individuals with MDD at high risk of suicide [44,45,46].

Additionally, repeated treatment with ketamine resulted in a more intense and prolonged response in patients with TRD [47].

Several mechanisms likely responsible for ketamine’s unique antidepressant effects have been identified, although not all are yet fully understood. To truly understand ketamine’s antidepressant effects and expand its clinical use, it is crucial to explore its molecular mechanisms in depth. This knowledge could help in developing more precise and effective psychiatric therapies, reshaping the current approaches to mental health treatment by identifying pathways that allow for greater accuracy and impact in care.

## 2. Materials and Methods

The aim of our study was to provide a narrative review of the latest findings on the molecular mechanisms underlying the antidepressant effects of ketamine in the treatment of depression. Our primary focus was on its NMDA receptor antagonism, effects on GABAergic interneurons, activation of AMPA receptors, the mTOR pathway, and interactions with BDNF. Additionally, we explored ketamine’s influence on neural pathways and highlighted the clinical implications arising from its molecular mechanisms.

It should be noted that our work is not systematic and is a narrative review aimed at a general assessment of the topic. By reviewing the selected articles, we have tried to broadly describe how ketamine demonstrates its antidepressant effects and answer the following questions:

1. What molecular mechanisms underlie the rapid antidepressant effects of ketamine?
2. What changes in neuronal networks are induced by ketamine, and how do they contribute to its therapeutic efficacy in depression?
3. What molecular mechanisms influence therapeutic approaches to using ketamine in MDD, including its impact on treatment personalization and efficacy optimization?

For the initial search, three authors independently searched the PubMed and ResearchGate database, which included works exclusively in English, only original data, from the years 1992 to 2024*. During the search, we used the keywords combined from “esketamine”, “ketamine”, “depression”, “molecular mechanism”, ”MDD”, “TRD”, “monoaminergic antidepressants”; “suicide”, “major depressive disorder”, “antidepressants drugs”, “sex differences”, ‘metabolism”, “pharmacology”, “Arketamine”, “synaptic remodeling”, “glutamatergic signaling”, “excitotoxicity“, “NMDA”, “structure”, “BDNF”, “neuroplasticity”, “TrkB”, “HCN1”, “GLT-1”, “GFAP”, “EAAT1”, “EAAT3”, “dlPFC”, “ACC”, “PFC”, “hippocampus”, “AMPA receptors”, “MRI”, “fMRI”, “neural network reorganization”, “structural changes”, “polymorphism”, “opioid receptors”, “alterations”, “DMN”, “CEN”, “SN”, and “functional connectivity”.

**Figure 1:**
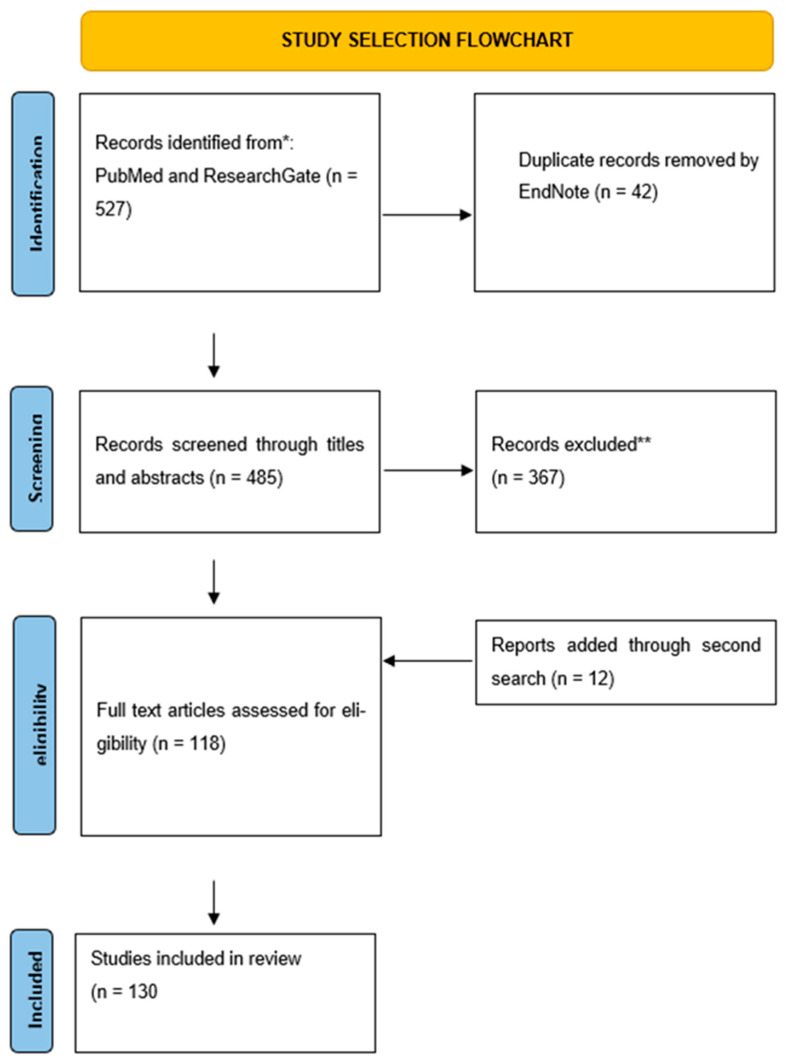
Flowchart of search strategy. * Due to frequent gaps in the PubMed database regarding selected molecular aspects, we included older studies if, after consultation between three authors, the study was deemed crucial (three authors indicated “yes” during the inclusion voting (“yes” or “no”). Additionally, we used ResearchGate for topics that were not sufficiently covered by data exclusively from PubMed. ** In this step, despite excluding studies that did not meet our inclusion criteria, we removed duplicate studies missed by the automated tool.

The search process is schematically illustrated in a flowchart (Figure 1). The database review was conducted in September 2024, and we collected 527 records. We removed duplicates using EndNote version 21 software. The second search was conducted in the same way in November and December 2024.

Next, the prepared database was reviewed by examining titles and abstracts. The review was conducted independently by three authors. We excluded works unrelated to our research questions and Preprint studies. Through a second search we added 12 studies. Finally, our narrative review included 130 studies. In addition, we extracted data regarding the study characteristics (authors, year, study design), the intervention used, population details, key findings, and limitations from studies on the subsection “Triple Network Dysfunction in Depression and Ketamine’s Influence”.

## 3. Pharmacology Profile of Ketamine

### 3.1. Structure

Ketamine (2-chlorphenyl-2-methylamino-cyclohexanone) is a chiral compound consisting of two optical enantiomers: esketamine (S(+)-ketamine) and R-ketamine (R(-)-ketamine) (referred to as S-ketamine and R-ketamine later). Its molecular mass is 238 g/mol, and it has a pKa 7.5. Used as hydrochloride, ketamine is a slightly acidic aqueous solution with a pH range of 3.5–5.5. A mixture containing both enantiomers is referred to as racemic ketamine. In its free base form, ketamine is lipid-soluble, allowing it to cross the blood–brain barrier rapidly [48].

### 3.2. Routes of Administration and Bioavailability

Ketamine, including its racemic mixture and enantiomers, esketamine and R-ketamine, is available in various formulations, with bioavailability differing based on the route of administration. Intravenous (IV) administration offers the highest bioavailability, reaching 100%. Intramuscular (IM) administration also demonstrates similarly high bioavailability. In the case of intranasal (IN) esketamine, bioavailability is estimated to be between 25% and 50%. However, oral administration is associated with relatively low and highly variable bioavailability, ranging from 10% to 20% [49,50,51].

### 3.3. Dosages and Time of Action

The most common and well-studied route is IV administration, where a subanesthetic dose of 0.5 mg/kg (range to 1 mg/kg) over 40 min has been shown to produce rapid antidepressant effects, with benefits lasting up to a week. The dosages used in studies for IM administration are similar to those used for IV administration [52,53,54].

Both IV and IM routes demonstrated high response rates, with peak effects observed approximately 24 h post-treatment in a pilot study [55]. Subcutaneous dosages are 0.1 to 0.5 mg/Kg of racemic ketamine and 0.5–1 mg/Kg of esketamine, with administration times similar to those used for IV in both cases [52,53,54].

IN ketamine, particularly of esketamine at doses ranging from 28 to 82 mg doses, has been shown to provide rapid antidepressant effects. The onset of action typically occurs within 5–40 min, with peak effects observed around 40 min post-administration [56,57]. The duration of action for intranasal ketamine is generally reported to last approximately from 2 to 4 h up to 28 days in some cases [58].

IM racemic ketamine administration resulted in reduced depression and anxiety ratings within 1 h of dosing, with these effects persisting for up to 7 days. Compared to intravenous infusion, IM administration offers several advantages, including reduced staff time and less equipment, making it a more efficient option for clinical practice [59].

SC racemic ketamine’s and esketamine’s antidepressant effects can last significantly longer than their pharmacokinetic half-life. The effects persist for days to weeks after administration, with repeated doses extending the duration of these effects [52]. Repeated doses, such as those administered weekly, can extend the duration of antidepressant effects, with some studies showing sustained improvements over six weeks [60].

Oral ketamine exhibits a prolonged elimination half-life, ranging from 7 to 9 h in controlled-release formulations, which is longer than in immediate-release forms [61].

The antidepressant effects of oral ketamine typically begin within 1–2 h after administration, with peak effects occurring later and sustained benefits lasting over two weeks in some cases [62].

In a study with prolonged-release ketamine, the antidepressant effects were observed over 14 days, although statistical significance was not achieved due to trial limitations [63].

In summary, the duration of ketamine’s antidepressant effects varies based on the route of administration and dosage. For instance, a single IV infusion can produce effects lasting several days to weeks, depending on the individual and the treatment regimen [54,64]. Repeated IV infusions have been shown to prolong the duration of the antidepressant response, with some studies indicating sustained effects for up to 15–30 days following higher doses (1.0 mg/kg) [65,66]. In contrast, lower doses (0.2 and 0.4 mg/kg) may provide shorter effect durations, emphasizing the need for careful dose titration [65].

### 3.4. Distribution, Metabolism and Elimination

Ketamine exhibits low plasma protein binding, about 10% to 30%. Its central compartment volume approximates 70 L, while the steady-state distribution volume is approximately 200 L or 2.3 L/kg. Ketamine is primarily eliminated from the body through the kidneys. The clearance of ketamine ranges from 1000 to 1600 mL/min (or 12 to 20 mL/min/kg) and is comparable to liver blood flow, making it dependent on that flow. The elimination half-life of ketamine in humans is between 1.5 and 5 h [67,68]. Women’s clearance rates may be approximately 20% higher than men’s [68].

It is worth mentioning that obesity alters the pharmacokinetics of ketamine. The increased adipose tissue in obese individuals can lead to a larger volume of distribution for lipophilic drugs like ketamine, which may result in prolonged effects and altered therapeutic outcomes [69].

Additionally, ketamine is relatively contraindicated in patients with active liver disease due to its potential to cause liver injury, particularly with repeated administration. Caution should be exercised when considering ketamine therapy in this population [70].

**Figure 2:**
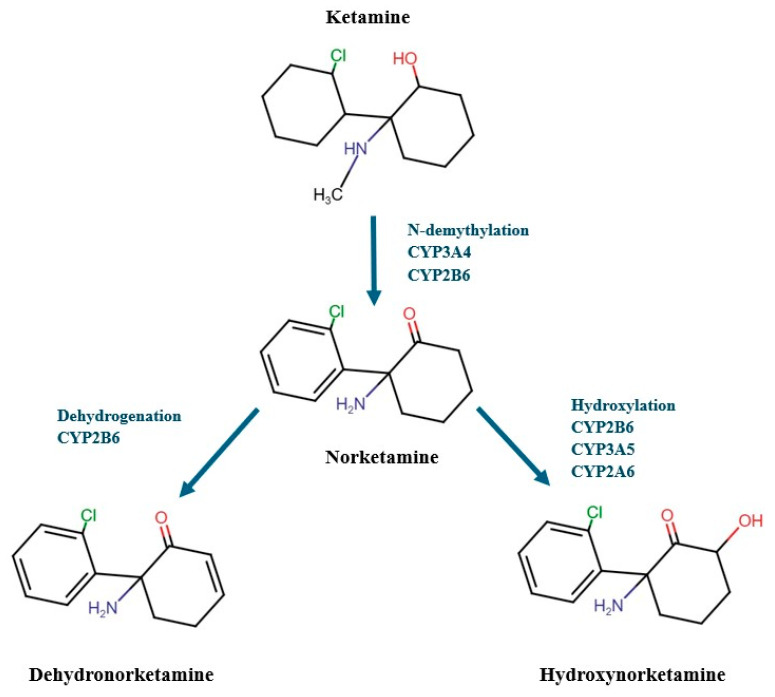
Pathway of 1 phase ketamine metabolism. Ketamine (both isomers) is metabolized through N-demethylation by liver cytochrome P450 enzymes (primarily by CYP3A4 and CYP2B6), resulting in the formation of norketamine. Norketamine is then mainly converted by CYP2B6, CYP3A5 and CYP2A6 through hydroxylation to hydroxynorketamine (**right path**) and by CYP2B6 through dehydrogenation to dehydronorketamine (**left path**).

Ketamine (both isomers) is primarily metabolized through N-demethylation by a microsomal enzyme system (hepatic biotransformation through the cytochrome P450 (CYP), primarily by isoenzymes 3A4 and 2B6), resulting in the formation of norketamine (Figure 2), which accounts for about 80% of the metabolites. Additional minor pathways, such as hydroxylation of the cyclohexanone ring of ketamine and norketamine, have been identified. Norketamine is then mainly converted by CYP2B6, CYP3A5, and CYP2A6 through hydroxylation to hydroxynorketamine (HNK) and by CYP2B6 through dehydrogenation to dehydronorketamine (DHNK) (Figure 2), which are eventually excreted in bile and urine following glucuronidation. Glucuronidation is primarily catalyzed by uridine-5′-diphospho-glucuronosyl-transferases (UGTs). Moreover, besides CYP2B6 and CYP3A4, CYP2C19 and CYP2D6 also play essential roles in ketamine metabolism, while CYP2C9’s involvement is considered insignificant [67,71,72,73,74].

Norketamine and HNK are enantiomerically selective, pharmacologically active metabolites that potentially contribute to the antidepressant and analgesic properties of ketamine [75].

The metabolism and disposition of ketamine exhibit stereoselectivity. Studies using human liver microsomes have shown that the N-demethylation of S-ketamine is 20% more efficient than that of R-ketamine and 10% more than the racemic mixture. Additionally, there is a metabolic enantiomeric interaction, where one enantiomer can inhibit the metabolism of the other [76]. Clinically, this is observed as R-ketamine reducing the systemic clearance of S-ketamine [77].

As mentioned above, ketamine is metabolized into (R, S)-norketamine. Subsequently, (R, S)-norketamine can be transformed into either (R, S)-DHNK or (R, S)-HNK. Notably, following ketamine administration, 12 different HNKs have been identified in human plasma, classified based on the location of the hydroxyl group on the cyclohexyl ring (at positions 4, 5, or 6) and their stereochemical configuration at two stereocenters (R, R; S, S; R, S; or S, R). Norketamine, DHNK, and specifically (2R,6R;2S,6S)-HNK can be detected in human plasma as early as 40 min after receiving an antidepressant dose of ketamine (0.5 mg/kg intravenously over 40 min), with (2R,6R;2S,6S)-HNK and (2S,6R;2R,6S)-HNK being the most prevalent HNKs observed [78,79,80,81].

## 4. NMDARs Mechanism

NMDARs are classified as glutamatergic ion channels that are composed of three different subunits that may be derived from seven subunit genes: *GluN1*, *GluN2A-D*, and *GluN3A-B* [82].

The NR1 subunit is essential for neurodevelopment and is expressed uniformly throughout the brain. In contrast, NR2 subunits exhibit region-specific and developmentally regulated expression patterns. NR2A is predominantly expressed in the neocortex and hippocampus, whereas NR2B is primarily localized to the forebrain and is the most common NR2 subunit in the mammalian central nervous system. Furthermore, levels of NR2B receptors decrease in the prefrontal cortex (PFC) of individuals diagnosed with MDD. In contrast, NR2C and NR2D subunits show high expression in the cerebellum and diencephalon/lower brainstem. Additionally, NR3 subunits are developmentally regulated and expressed in the neocortex, cerebellum, and hippocampus. These subunits have distinct isoforms due to the presence of alternative splicing. However, the functional relevance of the different splice forms remains uncertain [83,84,85].

NMDARs contribute to both long-term potentiation (LTP) and long-term depression (LTD). NMDARs containing NR2A subunits promote LTP, while NR2B subunits promote LTD [86].

NMDA receptors are tetrameric protein complexes, typically composed of two NR1 and two NR2 subunits, with NR3 subunits occurring less frequently. They encompass an ion channel that regulates the neuronal influx of calcium (Ca^2+^) in addition to sodium (Na^+^) influx and potassium (K^+^) efflux. These receptors are distinct in their dual gating mechanism, requiring ligand and voltage activation to allow ion flow. The ligand gate opens when the receptor binds two ligands: glutamate, the primary agonist, and either glycine or D-serine, the co-agonist [87]. The depolarization, followed by activation of other receptors, such as binding of glutamate to alpha-amino-3-hydroxy-5-methyl-4-isooxazole-propionic acid receptors (AMPARs), is crucial to open the voltage gate by expelling magnesium (Mg^2^⁺) ions, which initially block the NMDA channel pore [88]. This dual functionality endows NMDA receptors with the unique property of acting as “coincidence detectors”, enhancing excitatory transmission in response to converging synaptic signals [89].

In addition to their ligand and voltage gates, NMDA receptors possess multiple modulatory binding sites, including polyamine and zinc (Zn^2^⁺) binding sites. Activation of NMDA receptors leads to the engagement of second messenger systems, which are critical for regulating synaptic plasticity. Depending on the location of their activation—either synaptic or extrasynaptic—NMDA receptors can trigger neurotrophic pathways, such as those mediated by brain-derived neurotrophic factor (BDNF) or apoptotic pathways [87,88].

Ketamine, particularly S-ketamine due to its higher affinity for NMDA receptors, acts as a non-competitive channel blocker of NMDA receptors. It binds to human NMDA receptors by occupying a binding pocket in the central vestibule between the channel gate and the selectivity filter. The binding involves interactions with crucial amino acids, specifically leucine 642 on GluN2A and asparagine 616 on GluN1, which form hydrophobic and hydrogen-bond interactions with ketamine. Molecular dynamics simulations indicate that S-ketamine can move between two distinct locations within this binding pocket, contributing to its non-competitive channel-blocking action and subsequent antidepressant effects [90].

Interestingly, NMDARs are presented at both synaptic sites and extrasynaptic locations. The precise source of endogenous ligands at these extrasynaptic NMDARs remains unclear; however, potential contributors include glutamate spillover from synapses, glial release of glutamate, glycine, and D-serine, neuronal release of glycine and D-serine, and capillary extravasation of serum glycine [91].

Activation of extrasynaptic NMDARs by extracellular glutamate contributes to excitotoxicity and synaptic atrophy, processes closely associated with depression and other neuropsychiatric disorders. This understanding has led to the hypothesis that inhibiting NMDARs could promote synaptic regeneration and mitigate the adverse effects of depression and stress. Moreover, ketamine’s activity-independent blockade of NMDARs reduces phosphorylation of eukaryotic elongation factor 2 (eEF2), which, in turn, enhances BDNF expression and supports synaptic formation [92,93].

It also rapidly induces antidepressant effects by interrupting NMDAR-dependent burst activity in lateral habenula neurons [94].

However, whether this mechanism accounts for the drug’s long-term antidepressant effects remains unclear [95]. Another study demonstrated that ketamine, throughout the blockade of NMDA receptors, can quickly reverse behavioral deficits resulting from chronic stress, highlighting its potential as an effective antidepressant [96].

Miller et al. hypothesize that direct antagonism of NMDARs on pyramidal neurons (PNs) triggers a protein synthesis-dependent, cell-autonomous form of homeostatic synaptic plasticity, leading to an increase in excitatory synaptic input onto these neurons [97]. This effect is proposed to result from the selective blockade of NMDARs containing GluN2B subunits [98]. Furthermore, ketamine has been suggested to inhibit spontaneous neurotransmission through NMDARs, offering additional insights into its mechanism of action at the synaptic level [92,99].

Ketamine inhibits presynaptic NMDARs, leading to reduced activity of the presynaptic hyperpolarization-activated cyclic nucleotide-gated channel 1 (HCN1). HCN1, which mediates the hyperpolarization-activated current (Ih), plays a critical role in regulating several cellular functions, including excitability, dendritic integration, synaptic transmission, plasticity, and rhythmic activity in the central nervous system [100,101]. This decrease in activity subsequently enhances glutamate release and increases postsynaptic glutamate receptor activity. Inhibition of extrasynaptic NMDARs along with the concurrent activation of postsynaptic AMPA receptors facilitates the initiation of signaling pathways related to neuroplasticity [102].

This triggers an intracellular cascade involving mTOR, BDNF, TrkB, and the synthesis of synaptic proteins [103,104].

**Figure 3:**
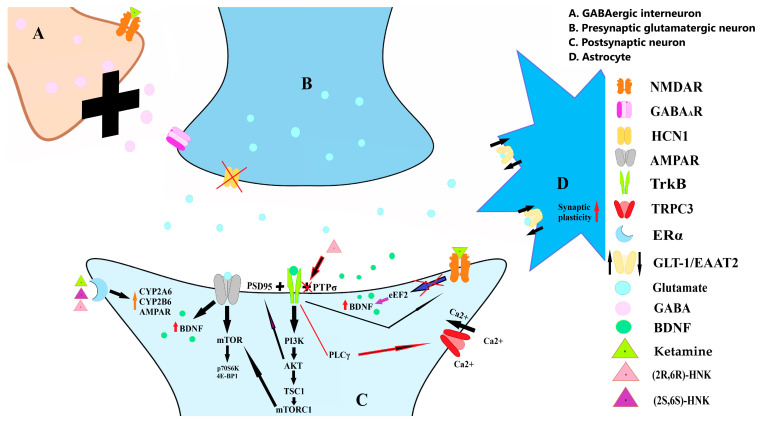
Ketamine blocks NMDA receptors on GABA-releasing interneurons (**A**), reducing GABA release from presynaptic glutamatergic neuron (**B**). Reduction in HCN1 activity promotes increased glutamate release. Glutamate activates AMPA receptors at a postsynaptic neuron (**C**), which initiates a cascade of intracellular events involving the mTOR pathway, elevating BDNF levels. The activation of mTOR is linked to increased phosphorylation of the proteins 4E-BP1 and p70S6K. Elevated BDNF levels activate TrkB receptors, activating TRPC3 through the TrkB-PLCγ pathway. Additionally, BDNF indirectly triggers rapid activation of the PI3K-Akt pathway, stabilizing the interaction between PSD-95 and TrkB to facilitate synaptic connectivity. TrkB modulates NMDA receptor activity by dissociating RasGrf1 from NR2B, reducing NMDA receptor signaling associated with LTD. Blockade of NMDA receptors also reduces phosphorylation of eEF2, which, in turn, enhances BDNF expression. Akt activation further influences cellular processes by inhibiting the TSC2 protein, releasing mTORC1 from regulatory constraints, and enhancing mTORC1 signaling. (2R,6R)-HNK dissociates TrkB from PTPσ, restoring juvenile-like plasticity in the brain. Additionally, ketamine administration restores GLT-1 levels in astrocytes (**D**), supporting glutamate reuptake and regulation. Activation of the ERα receptor initiates a positive feedback loop in which ketamine enhances the effects of its metabolites by increasing the activity of the enzymes CYP2A6 and CYP2B6.

Li et al. showed that ketamine rapidly activates the mTOR signaling pathway in rats’ PFCs. This activation involves increased phosphorylation of essential proteins associated with synaptic activity, including eukaryotic initiation factor 4E binding protein 1 (4E-BP1), p70S6 kinase (p70S6K). Notably, this phosphorylation is temporary, peaking shortly after ketamine administration and returning to baseline within 2 h. The effects were observed at low doses (5–10 mg/kg) but not higher anesthetic doses. Interestingly, blocking mTOR signaling completely prevents ketamine from inducing synaptogenesis and its antidepressant-like behaviors in depression models, highlighting the pivotal role of mTOR’s in ketamine’s rapid antidepressant effects. The study suggests that ketamine blocks NMDA receptors on GABA-releasing interneurons, reducing GABA release, which leads to disinhibition of glutamate signaling, activating the mTOR pathway (see Figure 3) [105].

Moreover, studies in animal models have shown that ketamine increases the ratio of AMPA receptors to NMDA receptors in the hippocampus, which may be crucial for its antidepressant effects. Chronic ketamine administration appears to exert its effects by modifying the composition of AMPA receptor density on the postsynaptic membrane [106,107]. This aligns with research conducted by Zanos et al., where (2R,6R)-HNK administration results in an initial increase in glutamatergic signaling, followed by a long-term upregulation of synaptic AMPARs in hippocampal synapses (substantiated by an elevation in the levels of GluA1 and GluA2) [108]. Interestingly, co-administration of alpha-amino-3-hydroxy-5-methyl-4-isooxazole-propionic acid (AMPA) and ketamine, each at very low doses that are individually ineffective, resulted in a significant antidepressant effect. Notably, these low doses are sufficient to minimize potential adverse effects typically associated with higher doses of either drug when used alone [109].

Moreover, ketamine exposure elicited messenger ribonucleic acid (mRNA) expression of the AMPA receptor subunit genes (*GRIA1*, *GRIA2*, and *GRIA4*). Furthermore, findings from this investigation also indicated the presence of a positive feedback loop through which estrogen can enhance the effects of ketamine and its (2R,6R)-HNK and (2S,6S)-HNK metabolites on the transcription of estrogen receptor alpha (ERα). Given the interaction of ketamine and its metabolites with ERα, one inquiry is whether there may be any potential apprehensions concerning estrogen-related neoplasms. The primary concern with unopposed estrogens appears to be the risk for endometrial carcinoma, which may suggest that monitoring postmenopausal women administered ketamine for endometrial irregularities would be judicious [110]. Further research on mechanisms of ketamine involving AMPARs in the treatment of MDD/TRD is needed.

### Sex Differences in NMDARs Expression and Their Influence on Ketamine’s Action

Glutamate levels and NMDA receptor expressions show notable sex-specific variations. Men tend to have higher glutamate levels in the prefrontal cortex, a region associated with executive functions [111]. In contrast, women exhibit elevated glutamate concentrations in the striatum and cerebellum, which are linked to reward and motor processing [112,113].

Additionally, the expression of NMDA receptor subunits, such as NR1 and NR2B, is more pronounced in females, particularly in the hippocampus and amygdala, regions critical for emotion and stress regulation [114]. These differences are further influenced by changes in estrogen levels throughout the menstrual cycle, which can affect the glutamate system and the functioning of NMDA receptors. Additionally, fluctuations in estrogen during the menstrual cycle may contribute to periodic variations in women’s vulnerability to MDD [115,116,117].

Interestingly, acute stress induces a more significant increase in glutamate release and NMDA receptor activation in women, potentially leading to excitotoxicity and impaired neuroplasticity. This may underlie women’s heightened vulnerability to stress-related disorders like MDD [118,119].

Sex differences in NMDA receptor signaling may also impact the efficacy and tolerability of NMDA-targeting antidepressants, including ketamine, which could be beneficial for specific therapy for MDD [119].

The estrous cycle significantly influences ketamine’s efficacy in female mice. During the follicular phase, when estradiol levels are low, ketamine’s effects are transient and weaker compared to the luteal phase, where estradiol (active form of estrogen) levels are high [120]. Estradiol modulates NMDA receptor density and activity in rat hippocampus, which may explain the differential response to ketamine across the estrous cycle [121,122]. The GluN2A subunit of NMDA receptors on parvalbumin (PV) interneurons is crucial for ketamine’s rapid antidepressant effects [120].

The differential expression of NMDA receptor subunits and the modulation by gonadal hormones provide mechanistic insights into why ketamine’s antidepressant effects may vary between sexes and across the female rodents’ estrous cycle. Preclinical studies show that female rodents are more sensitive to the antidepressant-like effects of low-dose ketamine compared to males. This enhanced sensitivity in females is mediated by ovarian hormones, particularly estrogen and progesterone [123]. Clinically, the rapid antidepressant response to ketamine does not differ significantly between men and women. However, repeated high doses of ketamine can induce adverse effects like anxiety and depressive behaviors more readily in females than in males [124].

Overall, the sex-specific effects of ketamine highlight the importance of considering biological sex as a critical factor when using ketamine as a rapid-acting antidepressant. Tailoring ketamine administration and dose based on sex and hormonal status may optimize therapeutic benefits while minimizing risks [125].

## 5. Neuroplasticity

### 5.1. BDNF Role in Depression

The disorders mentioned above may be related to BDNF, a neurotrophin responsible for neuronal survival, development, and synaptic plasticity. Altered BDNF levels are frequently observed in individuals with MDD, potentially contributing to the symptoms associated with the disorder. Decreased BDNF levels commonly lead to depressive symptoms through progressive neuronal loss and cortical atrophy, as BDNF typically elevates synapse quantity and spine density (e.g., in the apical dendrites of CA1 pyramidal neurons). Both pharmacological and non-pharmacological antidepressant therapies have been shown to help restore BDNF levels, suggesting a link between BDNF and the therapeutic effects of these treatments, which may promote neuroplasticity and recovery from depressive symptoms [126,127].

### 5.2. Ketamine’s Impact on BDNF and Its Pathways

In the context of treatment-resistant depression, BDNF may play a crucial role in response to novel antidepressant treatments such as ketamine. However, studies on ketamine’s effects on BDNF levels have produced mixed results. In some cases, ketamine and esketamine administration did not correlate with changes in serum or plasma BDNF levels, while other studies found that ketamine significantly increased plasma or serum BDNF [128,129,130,131,132,133,134,135].

In the study by Caliman-Fontes et al., a single dose of 0.5 mg/kg ketamine and 0.25 mg/kg esketamine was administered to patients with TRD. The results showed no significant changes in BDNF levels at the post-infusion evaluation points, and no differences were observed in BDNF levels between the two drugs. Despite both ketamine and esketamine exhibiting similar therapeutic effects, there was no correlation between BDNF levels and either treatment response or the severity of depressive symptoms. The study had limitations concerning the post-infusion assessment times (24 h, 72 h, and 7 days after administration), which may not have been optimal for measuring peripheral BDNF. The authors suggest that shorter intervals after ketamine infusion may be more suitable for detecting changes in BDNF [130].

On the other hand, the study by Jiang et al. showed that a small dose of esketamine, administered via micropumping in patients with post-partum depression, increased the serum concentration of 5-HT, DA, and BDNF compared to a placebo group. The dose used in this study was 0.25 mg/kg of esketamine, and serum BDNF levels were compared between pre-administration and the third-day post-administration [131].

Similarly, another study observed increased plasma BDNF levels 240 min after a 40-min IV infusion of 0.5 mg/kg ketamine. However, this study was limited by a small sample size [132]. Furthermore, the increased level of BDNF in plasma may be linked to treatment [135]. It is important to note that Meshkat et al. propose that peripheral measurements of total BDNF may not be reliable indicators of treatment response in patients with TRD. This suggests that peripheral BDNF levels might not fully capture the underlying mechanisms linked to antidepressant effectiveness in TRD [136].

The potential effect of ketamine on BDNF increases is induced by indirectly blocking GABA-inhibitory interneurons, which reduces the release of GABA and leads to impaired inhibition of glutamatergic neurons. As a result, more glutamate activates postsynaptic AMPARs, which in turn increases levels of BDNF [36,37].

The elevation of BDNF levels exerts an influence on the TrkB receptor, which supports the conclusion that ketamine also has an indirect effect on TrkB. The advantageous effects of ketamine on symptoms of depression are considerably attenuated in the presence of a TrkB antagonist. The administration of ketamine resulted not only in an elevation of BDNF levels but also in an increased ratio of phosphorylated tropomyosin-related kinase B (p-TrkB) to TrkB proteins within the hippocampus, which the authors associate with its rapid antidepressant-like properties [137,138].

Ketamine reduced receptor-mediated spontaneous miniature excitatory postsynaptic currents (NMDAR-mEPSCs) in hippocampal neurons within minutes, indicating a rapid inhibitory effect on NMDA receptor activity. In addition, protein extracts from ketamine-treated neurons showed reduced phosphorylation of eEF2, which leads to increased BDNF translation [92]. This demonstrates that the rapid antidepressant effect of ketamine may be significantly related to the increase in BDNF.

Moreover, Casarotto et al. found that ketamine and its metabolite 2R,6R-hydroxynorketamine ((2R,6R)-HNK) can directly bind to TrkB in a cholesterol-dependent process [139]. Furthermore, it may enhance BDNF signaling, thereby promoting synaptic structural potentiating mechanisms [78].

Another study demonstrates that (2R, 6R)-HNK promotes the association between TrkB and PSD95, an essential protein for neuronal connectivity, over a brief treatment duration of three days [140]. BDNF also enhances spine formation in neurons through two distinct pathways. First, by activating transient receptor potential canonical subfamily 3 (TRPC3) channels with the TrkB-PLCγ (Phospholipase Cγ) pathway, BDNF boosts steady currents essential for hippocampal spine density. Another part of this pathway increases cyclic adenosine monophosphate (cAMP), helping TrkB link with PSD-95. BDNF also helps PSD-95 move into neuron branches through a different, rapid PI3K (Phosphatidylinositol 3-kinase)-Akt pathway, creating stable connections (see Figure 3). Together, these paths stabilize and mature glutamate-based connections, strengthening synapse growth and the brain [141]. Furthermore, TrkB modulates N-methyl-D-aspartate (NMDA) signaling via a dual mechanism. Talebian et al. indicate that TrkB dissociates Ras protein-specific guanine nucleotide-releasing factor 1 (RasGrf1) from NR2B, leading to diminished NMDA signaling linked to LTD (p38-MAPK). Additionally, the association of RasGrf1 with TrkB promotes neurite outgrowth and phosphorylated extracellular signal-regulated kinase (pERK) activation, which are crucial for learning and memory [86].

The advantageous effects of ketamine on depression symptoms are considerably attenuated in the presence of a TrkB antagonist. The administration of ketamine resulted not only in an elevation of BDNF levels but also in an increased ratio of phosphorylated tropomyosin-related kinase B (p-TrkB) to TrkB proteins within the hippocampus, which some studies associate with its rapid antidepressant-like properties [137,138,142].

Activated Akt triggers the phosphorylation of Tuberous Sclerosis Complex 2 (TSC2), thereby producing an inhibitory impact on its role. This inhibitory action subsequently liberates mTOR Complex 1 (mTORC1) from its regulatory constraints. mTORC1 then phosphorylates downstream targets including p70S6K and 4E-BP1 (see Figure 3) [105,143].

Results from another study by Casarotto et al. demonstrate that ketamine and (2R, 6R)-HNK interfere with the TrkB and the protein tyrosine phosphatase sigma (PTPσ) interaction. PTPσ are predominantly found in adult neurons. The TrkB-PTPσ interaction typically results in TrkB dephosphorylation, which inhibits it signaling pathways (see Figure 3). By dissociating TrkB from PTPσ, ketamine, and (2R, 6R)-HNK facilitates a reestablishment of juvenile-like plasticity in the brain, enabling ocular dominance shifts while preserving the integrity of perineuronal nets around parvalbumin interneurons [144].

### 5.3. Glial Involvement

GLT-1 regulates synaptic glutamate levels, and its downregulation is linked to depressive behaviors and neuroplasticity deficits [145]. In a study by Fullana et al., a single dose of ketamine restored GLT-1 levels in knockdown mice to match controls. Blocking astroglial GLT-1 in the infralimbic cortex caused overactivity in glutamatergic neurons and deficits in the GABA system in mice, which researchers suggest may weaken neuronal circuit integrity and plasticity in MDD patients [146]. Additionally, in a separate study, Ardalan et al. found that ketamine modulates several structural parameters related to astroglial plasticity in the hippocampus within 24 h. The study supports the idea that impaired astrocytic morphology, which includes cell size and branching patterns in the hippocampus, contributes to depression [147,148].

GFAP is the central intermediate filament in astrocytes, providing mechanical strength and maintaining the cytoarchitecture of these cells, which is essential for the stability of the blood–brain barrier [149]. GFAP plays a crucial role in modulating glutamate transport by regulating both astrocytic (EAAT1) and neuronal (EAAT3) glutamate transporters. Its loss results in a 25–30% reduction in glutamate uptake in cortex and hippocampus [150]. In MDD, consistent pathology shows reduced expression of GFAP and altered astrocyte function, affecting glutamate uptake and synaptic development [151].

Disruption of normal GFAP function through aggregation can impair astrocytes’ glutamate buffering capacity, impacting neighboring neurons [152].

This is particularly relevant as rodents subjected to chronic unpredictable stress show reduced GFAP-positive cells in significant hippocampal areas (dentate gyrus, CA1, and CA3), alongside diminished spine density and lowered levels of BDNF, phosphorylated CREB, GLT-1, and PSD95 proteins in the hippocampus. Ketamine administration alleviates these stress-related deficits through BDNF-TrkB signaling-mediated regulation of GLT-1 in astrocytes [142].

Moreover, after ketamine administration, glutamate rapidly activates astrocytes, raising intracellular Ca^2^⁺ concentrations and significantly influencing synaptic plasticity. Ketamine’s effects on astrocytes may enhance the synaptic microenvironment, neurogenesis, and vascularization, thereby facilitating its rapid antidepressant action [153].

### 5.4. Structural Changes in Depression

A series of magnetic resonance imaging (MRI) studies have shown a reduction in the volume of both the PFC and hippocampus by up to about 19% in patients with MDD [78,154,155], and positron emission tomography (PET) reduced metabolism in PFC [78,156].

Moreover, lower synaptic density was found in the dorsolateral prefrontal cortex (dlPFC), anterior cingulate cortex (ACC), and hippocampus using PET with radioligand glycoprotein 2A synaptic vesicles [78,157].

The number of spinal synapses is also reduced in the PFC in MDD subjects [158,159].

Postmortem analyses have also observed reduced neuronal size and thickness of the cerebral cortex, especially in the medial and orbital areas in the PFC [159,160], and fewer granule cells in the dentate gyrus of the hippocampus in depressed individuals [161,162].

The above findings indicate that atrophy and neuronal loss in the PFC and hippocampus are significant cellular deficits in the pathogenesis of depression [104,159].

Furthermore, in human studies, 65 min after S-ketamine administration, all hippocampal subfields in healthy subjects showed increased volumes compared to placebo, with the most significant effect observed in the right hippocampal CA1 region [163].

Treccani et al.’s study on rodents showed that ketamine restored dendritic spines in the hippocampal CA1 region within 1 h of administration [164]. Twenty-four hours after a single ketamine injection, Ardalan et al. observed increased excitatory synapses in the CA1.SR area of the hippocampus in rats. These results support the idea that the rapid volumetric changes following ketamine treatment relate to enhanced vascularization in the hippocampus [165]. Moreover, Moda-Sava et al. highlighted that ketamine not only reversed dendritic spine loss but also rejuvenated disrupted microcircuits in the PFC, restoring both structure and function in this critical region affected by chronic stress [166].

### 5.5. Genetic Considerations

Interestingly, the BDNF *Val66Met* polymorphism blocks ketamine’s antidepressant effect in mice [167].

This polymorphism occurs when there is a substitution from valine to methionine at codon 66 in the BDNF gene (*Val66Met*), blocking the processing of the brain-derived neurotrophic factor precursor (proBDNF) to mature BDNF and its activity-dependent release. Approximately 30% of the U.S. population carries this polymorphism, which has been associated with increased vulnerability to stress-dependent behavior [168]. In contrast, Hosang et al. study showed no interactions between BDNF *Val66Met* polymorphism and life stress in depression [169]. In conclusion, polymorphism may interfere with the mechanisms driving ketamine’s antidepressant effects through enhancing BDNF, but more research on this topic is needed [168].

## 6. Opioid Receptor System Involvement in Ketamine Antidepressant Effect

Recent research suggests that ketamine’s antidepressant effect may also involve the opioid receptor system [170], which includes mu (MOR), kappa (KOR), and delta (DOR) receptors. This involvement is evidenced by the attenuation of ketamine’s antidepressant effects when opioid receptor antagonists are administered, indicating a potential interaction between ketamine and the opioid system [171]. Klein et al. demonstrated that while functional opioid receptors are necessary for ketamine’s antidepressant effects in rodents, they serve a permissive rather than direct role. This was evidenced by the fact that opioid receptor antagonists blocked ketamine’s antidepressant effects, yet activation of µ-opioid receptors (MOR) alone (via morphine) did not reproduce ketamine’s antidepressant-like effects despite producing hedonic responses. The researchers found that when ketamine was administered at doses effective for treating depression-like behaviors, it did not produce the hedonic effects characteristic of drugs that directly stimulate MOR. Their findings suggest that ketamine’s therapeutic action depends on both NMDA and opioid receptor signaling, not as a direct opioid agonist [172]. Zhang et al. found that pretreatment with naltrexone (10 mg/kg, administered 0.5 h prior) did not inhibit the acute (3-h) or sustained (1 to 2 days) antidepressant-like effects of (R, S)-ketamine (10 mg/kg) in mouse models of depression induced by chronic social defeat stress and inflammation. These findings suggest that the opioid system might not be involved in the antidepressant effects of ketamine in mice displaying depression-like behavior [173]. In contrast, research conducted on rodents revealed that inhibiting opioid receptors with naltrexone declined ketamine-induced neurophysiological alterations and synaptic adaptability in male rodents, signifying an essential function of opioid signaling in the antidepressant therapeutic effectiveness of ketamine. Interestingly, female rats showed no significant differences in response to opioid blockade, which suggests possible sex dependency [174].

What is noteworthy is that Nolan et al. suggest that ketamine induces a rapid antidepressant response predominantly through its interaction with MOR opioid receptors. This is even more convincing given that the influence of ketamine was indeed nullified by naltrexone in their analysis [171].

Furthermore, an interaction of submicromolar concentrations of ketamine with endogenous opioid peptides (methionine-enkephalin, leucine-enkephalin, or dynorphin A17) elicited substantial synergistic effects, activating opioid receptors. The degree of synergy varied among different receptors and peptides. Notably, the ketamine metabolite 6-hydroxynorketamine displayed significant allosteric modulatory properties at μ-opioid receptors; this metabolite is recognized for its analgesic and antidepressant effects, yet it does not interact with glutamate receptors [175].

Further analysis of the interaction between ketamine and the opioid system is needed. Identifying the specific opioid receptors involved in mediating ketamine’s antidepressant effects is essential. Additionally, assessing whether naltrexone’s blockade of ketamine is dose-dependent requires examination.

All described earlier molecular mechanisms of ketamine’s action are briefly presented in the table (see Table 2).

## 7. Triple Network Dysfunction in Depression and Ketamine Influence

Alterations in the structure, function, and reciprocal activity of various brain areas, including cortico-limbic circuits [176], may underlie impaired mood regulation in MDD. In this section of the review, we focused on the effect of ketamine on the triple network dysfunction model of depression. Which includes hyperactivity within the Default Mode Network (DMN), linked to excessive rumination and negative self-focus [177], increased connectivity within the Central Executive Network (CEN), concurrently decreased connectivity between the CEN and DMN, connected with a failure to regulate emotional responses effectively [178,179], and decreased effective connectivity from the Salience Network (SN) to the DMN, which may impair the ability to shift attention away from negative stimuli [180].

### 7.1. Structural and Functional Organization of Brain Networks in Depression

#### 7.1.1. Default Mode Network

DMN primarily consists of the posterior cingulate cortex (PCC), mPFC, and the bilateral temporal parietal junction (TPJ) [181]. The PCC is frequently highlighted as a central hub within the DMN, playing a crucial role in integrating information across the network and supporting self-referential and memory-related processes [182].

#### 7.1.2. Central Executive Network

The main structures of the CEN include dlPFC, which is critical for working memory and executive functions. It is involved in maintaining and manipulating information, as well as in decision-making processes. Functional connectivity (FC) between the dlPFC and other regions, such as the dorsal anterior cingulate cortex (dACC) and the fronto-insular cortex (FIC), is associated with working memory performance [183].

Moreover, the right dlPFC and right caudate are part of the fronto-striatal connections within the fronto-parietal network, highlighting their role in executive control. The fronto-parietal network integrates information across different brain regions to support cognitive tasks [184].

#### 7.1.3. Salience Network

The Anterior Insula (AI) is a pivotal region within the SN that detects novel and salient stimuli across multiple modalities. It plays a critical role in integrating sensory and emotional information, and its activity is modulated by homeostatic, emotional, or cognitive factors [185,186]. The ACC is another central hub of the SN, involved in cognitive control and emotional regulation. It works in conjunction with the AI to coordinate responses to salient stimuli [187,188]. The amygdala is a subcortical region that interacts with the SN, particularly influencing the AI and ACC during emotional processing. It amplifies the processing of negative emotional stimuli and facility attentional resource allocation [189]. The SN also incorporates other subcortical regions, such as the hypothalamus, thalamus, ventral striatum, and specific brain stem nuclei, which support its role in responding to homeostatic demands [190]. FIC is a critical region within the SN that mediates interactions between the CEN and other networks, such as the DMN. It modulates cognitive performance and executive tasks [191]. Abnormalities of the ACC, including severe decreases in cortical thickness, have been highlighted in MDD [192], reduced glutamate levels [193], and lower synapse density [157].

In addition, the subgenual anterior cingulate cortex (sgACC) plays a unique role in the induction of the depressive state due to increased metabolic activity, but also in response to various forms of antidepressant treatment. A mechanism of down-regulation of several GABA-related genes has been found in the sgACC of patients with major depression, resulting in lower levels of GABA and potentially affecting dendritic somatostatin targeting interneurons [194,195]. Another part of the ACC, responsible for the expression of anhedonic behavior and impaired emotion processing, is the pregenual anterior cingulate cortex (pgACC) [196]. Reduced glial density and neuronal size have been observed in this area. Moreover, reduced genetic expression of enzymes that enable glial glutamate reuptake and its conversion to glutamine has been demonstrated, leading to lower glutamine levels in MDD patients [197]. It has been speculated that the development of MDD is closely related to dysfunctional FC of the ACC with the mentioned earlier subregions [198].

### 7.2. Ketamine’s Modulatory Effects on Triple Network Function

Chen et al. investigated how subanesthetic doses of ketamine influence PFC-related circuits, focusing on the connectivity patterns within crucial brain regions such as the dACC, dlPFC, and mPFC. These are critical hubs within the SN, CEN, and DMN. The study found that a 0.2 mg/kg dose led to a notable decrease in FC within the dACC and other frontal and parietal regions. Similarly, the 0.5 mg/kg group reduced the FC of the left dACC and right dlPFC with other frontal regions. Interestingly, there was a negative association between reduced suicidal ideation and decreased FC between the left dACC and right ACC in the standard dose group. The low dose showed a positive association between reduced suicidal ideation and increased FC between the right dlPFC and left superior parietal cortex [199].

In individuals with TRD, ketamine enhanced functional connectivity between the caudate and prefrontal areas–left dlPFC and right ventrolateral prefrontal cortex, related to cognitive functions and between the putamen and prefrontal regions–and pgACC and orbitofrontal cortex (OFC) linked to affective processes. In contrast, in healthy volunteers, connectivity in these frontal areas diminished following ketamine administration [200].

This may be related to the previously mentioned frontal asymmetry in patients’ brain activity with TRD [201].

In addition, Wise et al. revealed in their metanalysis that smaller volumes of grey matter in the right dlPFC and left hippocampus were more significant in patients with MDD than in controls [202].

Further research by Li et al. highlights a link between changes in Hamilton Depression Rating Scale (HDRS) scores and volume alterations in the right dlPFC among TRD patients after receiving low-dose ketamine infusions. They observed that patients with TRD who had less volume reduction in the right dlPFC were more likely to respond to ketamine infusions. These results indirectly indicate that low-dose ketamine infusion delays or normalizes the rate of dlPFC volume reduction associated with TRD [203,204].

Insular connectivity with the DMN exhibited normalization in individuals diagnosed with MDD two days after a ketamine infusion, particularly within the right hemisphere. This observed alteration was reversed after ten days and was not evident in placebo scans. Additionally, the analysis identified regions demonstrating enhanced connectivity within the precentral and postcentral gyri among the MDD cohort. The investigation also reported increased connectivity in the thalamus, occipital cortex, and PFC following ketamine administration. Connectivity alterations in the insula among subjects with MDD imply that ketamine may normalize the interplay between the DMN and SN [205].

The administration of ketamine in individuals diagnosed with MDD was linked to a reduction in activity within regions (mPFC, PCC, and the precuneus) identified as components of the DMN. Furthermore, the activation pattern observed in MDD participants post-ketamine infusion mirrored the activation seen in healthy controls following a placebo infusion, indicating a potential normalization of functional dynamics during emotional processing [206].

In line with these findings, Li et al. showed decreased FC between the PCC and dmPFC within the DMN 24 h post-infusion of 0.5 mg/kg ketamine. This reduction in FC was linked to glutamatergic changes in the pgACC, indicating that glutamate modulation is associated with these connectivity changes. The delayed connectivity effects are likely the result of acute neuronal changes induced by ketamine, leading to long-lasting network reconfigurations, even after the drug has left the system [207].

TRD patients exhibited reduced connectivity within fronto-striatal pathways, with two critical circuits showing impairment: the connection between the right superior frontal cortex and striatal regions associated with executive function and the pathway linking the right PCC with striatal areas involved in motor control. Notably, when patients showed initially weaker connectivity between their bilateral superior frontal cortex and the executive portions of the striatum, they tended to show more substantial improvements in depressive symptoms following administration of ketamine at 0.2 mg/kg [208].

Ketamine also impacts the CEN by enhancing FC within resting-state network hubs, such as the anterior cingulate and frontal gyrus. The disinhibition of pyramidal neurons may drive this increase in PFC connectivity due to NMDAR blockade on GABAergic interneurons, particularly in the PFC. Additionally, ketamine-induced changes in resting-state functional magnetic resonance imaging (RS-fMRI) signals include a reduction in connectivity between the CEN and SN seed regions with the calcarine fissure in the occipital lobe, which forms part of the primary visual cortex. The reduction in connectivity between the SN and the calcarine fissure correlates with negative symptoms, potentially serving as predictive biomarkers for the negative symptoms induced by ketamine [209].

Another study found that 0.2 mg/kg of ketamine was associated with a more significant increase in PFC activity than 0.5 mg/kg, supporting previous findings that the antidepressant action of ketamine occurs only in a small and low-dose range. Most importantly, neuroimaging data suggested that increased glutamatergic neurotransmission in the PFC was critical to the ketamine-specific rapid antidepressant effect [210].

After approximately two hours post-administration, ketamine produced changes in brain connectivity in patients with remitted depression. These changes included altered connectivity in the CEN and increased negative connectivity between the ACC and the amygdala. Notably, these effects occurred without concurrent symptom changes and at a time when ketamine’s active metabolites were nearly absent from the bloodstream [211].

Interestingly, Kopelman et al. identified a link between changes in diffusion tensor imaging (DTI), mean diffusivity (Δ-MD), and ketamine treatment response in depressed patients. Decreased Δ-MD in the left rostral paracingulate gyrus and the left amygdala correlated with improved depression outcomes in those treated with ketamine. Conversely, in the right and left hippocampus, increased Δ-MD predicted better depression scores among ketamine recipients [212].

A growing number of clinical studies support the neurotrophic hypothesis of depression, which emphasizes the role of the PFC and hippocampus, including the disruption of neurotrophic factors and synaptic connections [213,214,215].

According to this theory, chronic stress leads to a decrease in neuroprotective factors, resulting in damaged or impaired plasticity, neuronal atrophy, and a reduction in synapses and their function, especially in these critical structures [216,217,218].

In addition, the PFC affects the amygdala, which is responsible for the control of fear and anxiety, which is closely related to depression [219,220].

Ketamine has been shown to reduce the responsiveness of the hippocampus, mPFC, and PCC/precuneus complex during negative emotional processing. Interestingly, ketamine was found to play a significant role in moderating the relationship between mPFC activity and ruminative thinking. Lamotrigine administration was associated with counteracting ketamine’s enhancement of frontal–limbic connections in the acute phase. However, ketamine did not influence the modulation of the hippocampus, mPFC, or PCC/precuneus regions. In the long term, ketamine reduced PCC/cerebellar responses, which were prevented by lamotrigine due to its inhibition of glutamate release. These findings underscore ketamine’s role in suppressing limbic system reactivity [221].

Further studies highlighted ketamine’s influence on specific subregions within the ACC. Ketamine significantly altered sgACC-hippocampal, sgACC-ventromedial prefrontal cortex (vmPFC), pgACC-PCC, and dACC-supramarginal gyrus connectivity. Notably, changes in pgACC-insula connectivity were associated with improvements in depression scores, suggesting a link between connectivity alterations and symptom relief. Moreover, the sgACC was most substantially modulated by ketamine, with changes in sgACC-pgACC, sgACC-ventral striatal, and sgACC-dACC connectivity correlating with reductions in anhedonia symptoms [222].

24 h after ketamine administration, the glutamine/glutamate ratio in the pgACC was significantly elevated compared to placebo [223].

Finally, Gärtner et al. demonstrated that increased connectivity post-ketamine between the sgACC and right lateral PFC correlated with symptom reduction. Furthermore, the findings indicate that lower baseline connectivity before ketamine treatment between these regions is linked to more significant depressive symptom relief [224].

In summary, ketamine’s multifaceted modulation of brain connectivity across networks, particularly in areas tied to emotional and cognitive processing, appears to counteract critical neurobiological deficits in depression (for more detailed description see Table 3). These findings support ketamine’s therapeutic effects as well as its role in the neurotrophic hypothesis of depression, pointing to its impact on restoring network balance and enhancing resilience in neural circuits central to emotional regulation. Nevertheless, future research on ketamine’s modulation of brain connectivity is needed to deepen our understanding of its therapeutic mechanisms.

## 8. Conclusions

The search for fast-acting antidepressant drugs is critical to provide immediate protection against suicide risk while awaiting the effects of conventional treatments. Ketamine, with its unique receptor profile, emerges as a promising candidate in this context, though only its S-enantiomer (Spravato) has been approved for therapeutic use. Due to its narcotic nature, rapid potential for dependence, and significant side effects, ketamine’s clinical application is highly restricted, emphasizing the need for deeper investigation into its molecular mechanisms to optimize patient-specific benefits while minimizing potential risks.

Ketamine’s primary mechanism involves its action as a non-competitive NMDA receptor antagonist, particularly targeting receptors with the NR2B subunit. By inhibiting NMDA receptor activity on GABAergic interneurons, ketamine indirectly stimulates glutamate release and AMPA receptor activation at target synapses. This cascade triggers the BDNF-TrkB pathway, essential for neurogenesis and synaptic regeneration, especially in the prefrontal cortex and hippocampus, regions often affected by atrophy in depression. Ketamine also rapidly activates the mTOR pathway, further enhancing synaptic strength and density through increased AMPA receptor expression.

A potential therapeutic limitation lies in the BDNF polymorphism, which in certain studies correlates with a reduced antidepressant response in individuals carrying this variant [167]. In addition to directly affecting neurons, ketamine influences astrocytes and glutamate transport, which helps to maintain optimal synaptic conditions and prevent glutamate-induced neurotoxicity-effects particularly beneficial in the context of chronic stress. All described molecular mechanisms of ketamine’s action are presented in Table 2.

Ketamine’s action extends to several critical brain networks involved in emotional regulation and cognitive processing, including the Default Mode Network (DMN), Central Executive Network (CEN), and Salience Network (SN), which are frequently dysregulated in depression. By restoring balance and connectivity within these networks, ketamine enhances cognitive and emotional processing, contributing to the alleviation of depressive symptoms.

In summary, ketamine’s antidepressant effects result from its complex interactions across neurotransmitter systems, neural networks, and cellular pathways. These mechanisms underscore its therapeutic potential in major depressive disorder (MDD) and treatment-resistant depression (TRD), while highlighting the necessity for further research into its long-term efficacy and safety profile.

## Figures and Tables

**Table 1:** Summary of the most widely described receptor mechanisms of action of approved antidepressant drugs in depression treatment.

Groups of Antidepressants	Mechanism of Action	Example of a Drug	Reference
SSRI	Affinity for 5-HT2 and 5-HT1A receptors, inhibit serotonin transporter SERT	Fluoxetine Sertraline	[21,22]
MAOI	Monoamine oxidase enzyme inhibition	Selegiline Moclobemide	[23,24]
TCA	Blocking of presynaptic norepinephrine reuptake transporters and presynaptic serotonin reuptake transporters, antagonism of postsynaptic A1- and A2-adrenergic receptors, postsynaptic muscarinic receptors, postsynaptic histamine H1 receptors	Amitriptyline Desipramine	[24,25,26,27]
SNRI	Blocking of norepinephrine reuptake transporters and serotonin reuptake transporters, minimal or no effect on adrenergic (A1, A2 and Β), histamine (H1), muscarinic, dopaminergic or postsynaptic 5-HT receptors	Duloxetine Venlafaxine	[28,29]
NRI	Blocking of norepinephrine transporter NET	Reboxetine Nisoxetine	[20,29]
NDRI	Binding affinity to DA and NE transporters	Bupropion	[20]
SARI	Affinity for A1-adrenergic receptor	Trazodone Nefazodone	[30]
NASSA	Antagonism of presynaptic A2 adrenergic autoreceptors and 5-HT2, 5-HT3 receptors	Mirtazapine	[27,31]
MMA	Affinity for 5-HT receptors (5-HT1A, 5-HT1B, 5-HT3A, 5-HT7)	Vortioxetine Vilazodone	[33,34]
TeCA	Antagonism of presynaptic A2 autoreceptors	Mianserin	[32]
Other drugs	Agonism to the 5-HT1A receptor	Buspirone Tandospirone	[20,32,35]
	Antagonism of presynaptic A2 autoreceptors	Trazodone (SARI)	[32]
Dissociative anesthetics	Non-competitive NMDA receptor antagonist, activation of postsynaptic AMPA receptors	Ketamine	[36,37]

**Table 2:** Summary of collected data on the molecular mechanisms of action of ketamine and its metabolites.

Mechanism	Main Findings	Studies Used	Substance Used
NMDA Receptor Antagonism	Ketamine functions as a non-competitive antagonist of NMDA receptors, resulting in reduced GABA release, enhanced glutamate release, and the promotion of synaptic plasticity	Zanos et al. [108]	(2R,6R)-HNK
		Li et al. [105] Yang et al. [94] Li et al. [96] Miller et al. [98] Autry et al. [92]	Ketamine
AMPA Receptors	The activation of AMPA receptors by glutamate enhances synaptic signaling and initiates intracellular cascades, including the production of BDNF and the activation of the mTOR pathway. Ketamine increases the ratio of AMPA receptors to NMDA receptors.	Li et al. [105] Casarotto et al. [139] Tizabi [106] Ho [110]	Ketamine
		Zanos et al. [108] Casarotto et al. [139] Ho [110]	Ketamine (2R,6R)-HNK
		Ho [110]	(2S,6S)-HNK
mTOR Signaling	Ketamine activates mTOR signaling, which promotes synaptic protein synthesis and the formation of dendritic spines	Li et al. [105] Moda-Sava et al. [166]	Ketamine
BDNF-TrkB Pathway	Ketamine increases the expression of BDNF, which binds to TrkB receptors, thereby enhancing synaptic connectivity and structural plasticity	Caliman-Fontes et al. [130] Rybakowski et al. [134] Li et al. [137] Woelfer et al. [128] Casarotto et al. [139] Haile et al. [132] Chen et al. [133] Duncan et al. [135] Cannarozzo et al. [144]	Ketamine
		Casarotto et al. [139] Jiang et al. [131] Caliman-Fontes et al. [130] Luo et al. [129]	Esketamine
		Fred et al. [140] Casarotto et al. [139] Ju et al. [138] Cannarozzo et al. [144]	(2R,6R)-HNK
Glial Involvement	Ketamine restores GLT-1 levels in the infralimbic cortex	Fullana et al. [146] Liu et al. [142]	Ketamine
Opioid Receptor System Involvement	Ketamine interacts permissively with opioid receptors, facilitating its antidepressant effects without functioning as a direct opioid agonist	Klein et al. [172] Nolan et al. [101] Zhang et al. [173] Di Ianni et al. [174]	Ketamine

**Table 3:** An extended description of the effects of ketamine on Triple Network Dysfunction in depression.

Authors	Study Design	Intervention	Main Findings	Limitations
Gärtner et al. [224]	Observational study	0.5 mg/kg racemic or 0.25 mg/kg S-ketamine IV over 45 min	Changes in sgACC and right lateral PFC connectivity correlated with improved anhedonia symptoms.	- Lack of a placebo control group. - Differences between racemic and S-ketamine - Need for additional scanning time points to understand the persistence of ketamine’s effects
Li et al. [223]	Double-blind, randomized, placebo-controlled study	0.5 mg/kg racemic ketamine IV over 40 min	Ketamine administration 24 h after infusion specifically increased the glutamine/glutamate ratio in the pgACC but not in the aMCC.	- The study was conducted in healthy subjects. - Only the delayed effects were examined. - The quantification of metabolites like glutamine and glutamate may be affected by technical factors like baseline distortion and macromolecule contributions.
Alexander et al. [222]	Randomized, double-blind, placebo-controlled crossover trial	0.5 mg/kg ketamine IV over 40 min	Changes in subgenual ACC connectivity correlated with improvements in anhedonia symptoms.	- Small sample size. - Lack of cross-validation. - Limited to a single time point. - The integrity of participant blinding was not assessed, which could have confounded the clinical score improvements.
Meiering et al. [221]	Double-blind, single-dose, randomized, placebo-controlled study	(1) Ketamine infusion: 0.12 ± 0.003 mg/kg for the first minute, followed by a continuous infusion of 0.31 mg/kg/h for approximately 55 min. (2) Lamotrigine pretreatment: 300 mg oral dose 2 h before the scanning procedures.	Ketamine increased amygdala-PFC connectivity and reduced activity in the hippocampus, and mPFC.	- The PPI analysis used is correlational and does not allow conclusions about the direction of the fronto-limbic relationship. - The study was conducted in healthy subjects.
Kopelman et al. [212]	Randomized, double-blind, placebo-controlled trial	0.5 mg/kg ketamine IV over 40 min	Changes in a marker of neuroplasticity (DTI-MD) were associated with improvements in depression scores, particularly in the left BA10 and left amygdala regions for the ketamine group.	- The neuroplasticity (Δ-MD) measure is an indirect proxy, and other factors like inflammation could confound the interpretation. - The effect sizes linking Δ-MD to clinical improvement were small. - Ketamine did not have a primary effect on Δ-MD, suggesting neuroplasticity is not the sole mediator of its antidepressant effects.
Burrows et al. [211]	Double-blind, randomized, crossover, placebo-controlled study	0.5 mg/kg ketamine IV over 45 min	Ketamine produced significant changes in brain connectivity, including decreased connectivity between the sgACC and amygdala, as well as altered connectivity in the ECN, approximately 2 h after administration in volunteers with remitted depression.	- No healthy control group to confirm if effects are specific to remitted depression. - Unclear if effects are unique to remitted depression or general to ketamine. - Uncertainty about the link between early effects and later antidepressant response. - No fMRI task to assess the functional impact of connectivity changes.
Li et al. [210]	Randomized, double-blind, placebo-controlled, parallel-group clinical trial	0.5 mg/kg or 0.2 mg/kg of ketamine IV	The increase in PFC activity correlated with rapid antidepressant effects at 40- and 240-min post-treatment.	- Patients were not required to discontinue their current medications. - The HRDS scale may not be ideal for assessing short-term changes in depression. - The study focused only on initial effects, leaving the link between PFC activation and long-term outcomes unclear.
Mueller et al. [209]	Randomized, double-blind, placebo-controlled crossover trial	S-ketamine bolus (0.1 mg/kg) + continuous infusion (0.015625 mg/kg/min for up to 1 h, with a 10% dosage reduction every 10 min.)	Decreased SN connectivity linked to negative symptoms induced by ketamine. Ketamine increased FC in the ECN.	- Only male subjects. - Numerous sessions were missing or prematurely terminated. - The study design did not allow for determining the exact onset of ketamine-induced symptoms or measuring ketamine plasma levels. - The ketamine dosage used was low. - Important vital signs were not available for analysis.
Chen et al. [208]	Randomized, double-blind, placebo-controlled trial	0.5 or 0.2 mg/kg ketamine IV over 40 min	- Patients with TRD showed decreased FC in the fronto-striatal circuits, particularly between the right superior frontal cortex and the executive region of the striatum and between the right paracingulate cortex and the rostral-motor region of the striatum, compared to healthy controls.	- Small sample size for the placebo group. - Patients were on other medications during the ketamine infusion. - Using global signal regression in the fMRI analysis may have introduced artificial anti-correlations. - Inconsistency in interview modality (phone vs. in-person) for clinical assessments.
Li et al. [207]	Double-blind, randomized, placebo-controlled study	Single dose of ketamine 0.5 mg/kg over 40 min	Ketamine administration led to a decrease in FC between the dorsal posterior cingulate cortex and the dmPFC at 24 h post-infusion, which was correlated with an increase in the glutamine/glutamate ratio in the perigenual anterior cingulate cortex at the same time point.	- The study was conducted in healthy subjects. - Acute effects during infusion were not measured due to technical limitations. - Only two post-infusion time points were examined
Mkrtchian et al. [200]	Double-blind, placebo-controlled, crossover trial	0.5 mg/kg ketamine IV over 40 min	- The increased fronto-striatal connectivity observed in TRD participants after ketamine was associated with sustained improvements in anhedonia symptoms. - Ketamine increased fronto-striatal functional connectivity in individuals with TRD but decreased in HVs.	- The rsfMRI scan was conducted 2 days after the ketamine infusion, while ketamine’s beneficial effects peak around 24 h. - The sample size was limited for the analyses examining relationships between neural changes, symptoms, and inflammation, and these analyses were exploratory and should be considered preliminary.
Reed et al. [206]	Randomized, double-blind, placebo-controlled crossover trial	0.5 mg/kg ketamine IV over 40 min	- MDD participants showed lower brain activation post-ketamine than post-placebo, while healthy controls showed the opposite pattern. - Ketamine appeared to normalize brain activation in participants with MDD, such that their post-ketamine activation pattern resembled the activation pattern of healthy controls after placebo.	- Reduced sample size due to some participants needing more usable data for all scan sessions. - Use of saline solution as a placebo, which may not have been an ideal control due to the dissociative effects of ketamine. - Lack of a baseline condition for each drug condition.
Evans et al. [205]	Double-blind, placebo-controlled, crossover study	0.5 mg/kg of ketamine hydrochloride IV over 40 min	- In subjects with MDD, connectivity between the insula and the DMN was normalized compared to healthy controls 2 days after a single ketamine infusion, but this change was reversed after 10 days.	- Using a more lenient initial statistical threshold could have led to more false positives. - Data are missing due to subjects not completing all scans addressed using a statistical model. - Lack of a sensitive behavioral measure to capture mood changes in the healthy control group on day 2.
Chen et al. [199]	Double-blind, randomized, placebo-controlled, longitudinal study	0.2 mg/kg or 0.5 mg/kg of ketamine IV over 40 min	- Ketamine infusion, particularly at a low dose of 0.2 mg/kg, reduced the FC of the dACC with other frontal and parietal regions. - The reduction in suicidal ideation was negatively correlated with the decrease in FC between the left dACC and right anterior cingulate cortex in the standard-dose group and positively correlated with the increase in FC between the right dlPFC and left superior parietal region in the low-dose group.	- The fMRI scans were performed 48 h after ketamine administration, so the immediate effects may not have been captured. - Patients were not required to discontinue their current medications. - The use of the MADRS item 10 rather than a dedicated suicide scale limited the ability to assess suicidality fully.
Li et al. [203]	Double-blind, randomized, placebo-controlled trial	0.5 mg/kg single infusion of ketamine or 0.045 mg/kg midazolam	A smaller decrease in the right dlPFC volume was associated with a more significant reduction in depressive symptoms in the ketamine group.	- Patients were not required to discontinue their current medications. - Neuroimaging was only conducted at baseline and Day 3, which may have missed earlier changes in brain volume.

## Abbreviations

ACC	Anterior cingulate cortex.
AI	Anterior insula
AMPA	Alpha-amino-3-hydroxy-5-methyl-4-isooxazole-propionic acid
AMPARs	Alpha-amino-3-hydroxy-5-methyl-4-isooxazole-propionic acid receptors
Bcl2/Bax	B-cell lymphoma 2/Bcl-2-associated X protein
BDNF	Brain-derived neurotrophic factor
CA1.SR	CA1 stratum radiatum
Ca^2+^	Calcium
cAMP	Cyclic adenosine monophosphate
CEN	Central Executive Network
CNS	Central nervous system
CREB	Phosphorylated cAMP response element binding protein
CUS	Chronic unpredictable stress
CYP	Cytochrome P450
dACC	Dorsal anterior cingulate cortex
DA	Dopamine
DHNK	Dehydronorketamine
dlPFC	Dorsolateral prefrontal cortex
DMN	Default Mode Network
dmPFC	Dorsomedial prefrontal cortex
DOR	Delta
DTI	Diffusion tensor imaging
EAAT1	Excitatory amino acid transporter 1
EAAT3	Excitatory amino acid transporter 3
eEF2	Eukaryotic elongation factor 2
ERα	Estrogen receptor alpha
FC	Functional connectivity
FIC	Fronto-insular cortex
GABA	Gamma-aminobutyric acid
GFAP	Glial fibrillary acidic protein
GLT-1	Glutamate transporter 1
HCN1	Hyperpolarization-activated cyclic nucleotide-gated channel 1
HDRS	Hamilton Depression Rating Scale
HNK	Hydroxynorketamine
Ih	Hyperpolarization-activated current
IM	Intramuscular
IN	Intranasal
IV	Intravenous
K^+^	Potassium
KOR	Kappa
LTD	Long-term depression
LTP	Long-term potentiation
MAOIs	Monoamine oxidase inhibitors
MDD	Major depressive disorder
Mg^2^⁺	Magnesium
MMAs	Multimodal antidepressants
MOR	Mu
mPFC	Medial prefrontal cortex
MRI	Magnetic resonance imaging
mRNA	Messenger ribonucleic acid
mTORC1	mTOR Complex 1
mTOR	Mechanistic target of rapamycin
Na^+^	Sodium
NASSAs	Specific serotonergic antidepressants
NDRIs	NE reuptake inhibitors
NE	Norepinephrine
NET	Norepinephrine transporter
mEPSCs	Miniature excitatory postsynaptic currents
NMDA	N-methyl-D-aspartate
NMDARs	N-methyl-D-aspartate acid glutamate receptors
NRIs	Selective norepinephrine reuptake inhibitors
OFC	Orbitofrontal cortex
p70S6K	p70S6 kinase
PCC	Posterior cingulate cortex
pERK	Phosphorylated extracellular signal-regulated kinase
PET	Positron emission tomography
PFC	Prefrontal cortex
pgACC	Pregenual anterior cingulate cortex
PI3K	Phosphatidylinositol 3-kinase
PLCγ	Phospholipase Cγ
PNs	Pyramidal neurons
PrL	Prelimbic
proBDNF	Brain-derived neurotrophic factor precursor
PSD95	Postsynaptic density 95
PTPσ	Protein tyrosine phosphatase sigma
p-TrkB	Phosphorylated tropomyosin-related kinase B
RasGrf1	Ras protein-specific guanine nucleotide-releasing factor 1
R-ketamine	Arketamine
RS-fMRI	Resting-state functional magnetic resonance imaging
SARIs	Serotonin-2 antagonists and reuptake inhibitors
SC	Subcutaneous
SERT	Serotonin transporter
sgACC	Subgenual anterior cingulate cortex
S-ketamine	Esketamine
SNRIs	Norepinephrine reuptake inhibitors
SN	Salience Network
SSRIs	Selective serotonin reuptake inhibitors
TCAs	Tricyclic antidepressants
TeCAs	Tetracyclic antidepressants
TPJ	Bilateral temporal parietal junction
TRD	Treatment-resistant depression
TrkB	Tropomyosin receptor kinase B
TRPC3	Transient receptor potential canonical subfamily 3
TSC2	Tuberous Sclerosis Complex 2
UGTs	Uridine-5′-diphospho-glucuronosyl-transferases
*Val66Met*	Substitution from valine to methionine at codon 66
vlPFC	Ventrolateral prefrontal cortex
vmPFC	Ventromedial prefrontal cortex
WHO	World Health Organization
Zn^2^⁺	Zinc
Δ-MD	Changes i[111]n DTI mean diffusivity
μOR	μ-opioid receptor
4E-BP1	Eukaryotic initiation factor 4E binding protein 1
5-HT	Serotonin
